# Study on Neuroprotective Mechanism of Houshiheisan in Ischemic Stroke Based on Transcriptomics and Experimental Verification

**DOI:** 10.1155/2023/8673136

**Published:** 2023-02-06

**Authors:** Hongfa Cheng, Yawen Zhang, Xiaoyao Guo, Xuan Wang, Hanyu Wang, Hui Zhao, Lei Wang, Haiyan Zou, Qiuxia Zhang

**Affiliations:** ^1^School of Traditional Chinese Medicine, Capital Medical University, Beijing 100069, China; ^2^Beijing Key Lab of TCM Collateral Disease Theory Research, Beijing 100069, China

## Abstract

Houshiheisan (HSHS), a classic prescription in traditional Chinese medicine (TCM), has shown outstanding efficacy in treating stroke. This study investigated various therapeutic targets of HSHS for ischemic stroke using mRNA transcriptomics. Herein, rats were randomly separated into the sham, model, HSHS 5.25 g/kg (HSHS5.25), and HSHS 10.5 g/kg (HSHS10.5) groups. Rats suffering from stroke were induced by permanent middle cerebral artery occlusion (pMCAO). After seven days of HSHS treatment, behavioral tests were conducted, and histological damage was examined with hematoxylin-eosin (HE). The mRNA expression profiles were identified using microarray analysis and quantitative real-time PCR (qRT-PCR) validated gene expression changes. An analysis of gene ontology and pathway enrichment was conducted to analyze potential mechanisms confirmed using immunofluorescence and western blotting. HSHS5.25 and HSHS10.5 improved neurological deficits and pathological injury in pMCAO rats. The intersections of 666 differentially expressed genes (DEGs) were chosen using transcriptomics analysis in the sham, model, and HSHS10.5 groups. The enrichment analysis suggested that the therapeutic targets of HSHS might regulate the apoptotic process and ERK1/2 signaling pathway, which was related to neuronal survival. Moreover, TUNEL and immunofluorescence analysis indicated that HSHS inhibited apoptosis and enhanced neuronal survival in the ischemic lesion. Western blot and immunofluorescence assay indicated that HSHS10.5 decreased Bax/Bcl-2 ratio and suppressed caspase-3 activation, while the phosphorylation of ERK1/2 and CREB was upregulated in a stroke rat model after HSHS treatment. Effective inhibition of neuronal apoptosis by activating the ERK1/2-CREB signaling pathway may be a potential mechanism for HSHS in the treatment of ischemic stroke.

## 1. Introduction

Stroke is a significant cause of death and morbidity among adults. To date, 80% of stroke cases occur due to thromboembolic occlusion. Neuron death and brain atrophy following a sudden drop in regional cerebral blood flow can cause permanent neurological damage [1, 2]. Intravenous thrombolysis and mechanical thrombectomy are effective methods for ischemic stroke in clinics [3]. However, they have some application limitations, including a narrow therapeutic time window, strict evaluation criteria, and the risk of hemorrhage [4, 5]. Additionally, more than 80% of stroke survivors suffer from motor impairment of the upper extremities and 50% still have it four years after the stroke [6]. Therefore, neuroprotective strategies bring the greatest hope for stroke survivors, while neuronal protection and regeneration have been the main focus to effectively rescue functional brain deficits [7].

Traditional Chinese medicine (TCM) has a long history and unique advantage in treating ischemic stroke [8], which has been one of the essential sources for new drug development to treat ischemic stroke [9]. As the first classic prescription for stroke, HSHS can promote recovery of limb and language function in patients with ischemic stroke and improve clinically the quality of their lives [10, 11]. HSHS plays a neuroprotective effect by decreasing inflammatory factor expression [12] and reducing amyloid precursor protein accumulation 24 and 72 h after cerebral ischemia [13]. Additionally, HSHS improved axon growth by inhibiting Nogo-A/RhoA/ROCK2 and Netrin-1/rac1/Cdc42 pathways [14] and promoted angiogenesis by regulating HIF1*α*/VEGF and Ang-1/Ang-2 pathways to alleviate neurological damage after seven days of stroke [15]. However, the mechanisms and pathways underlying the multitargeted effects of HSHS on ischemic stroke have been incompletely elucidated.

Using advanced omics technology to study applying TCM is an efficient and comprehensive method that links traditional Chinese medicine and Western medicine [16, 17]. As an essential part of systems biology, transcriptomics technology is an effective tool to detect the expression changes of global RNA in corresponding proteins [18, 19]. Transcriptomics analysis can determine the precise therapeutic targets and their interactions, which is essential to clarify the multifaceted mechanism of traditional Chinese medicine prescriptions [20]. High-throughput RNA-seq and microarray analyses have been widely used to reveal molecular mechanisms of Chinese herbal medicines for diseases such as stroke, cancer, and hypertension [21–23].

**Figure 1:**
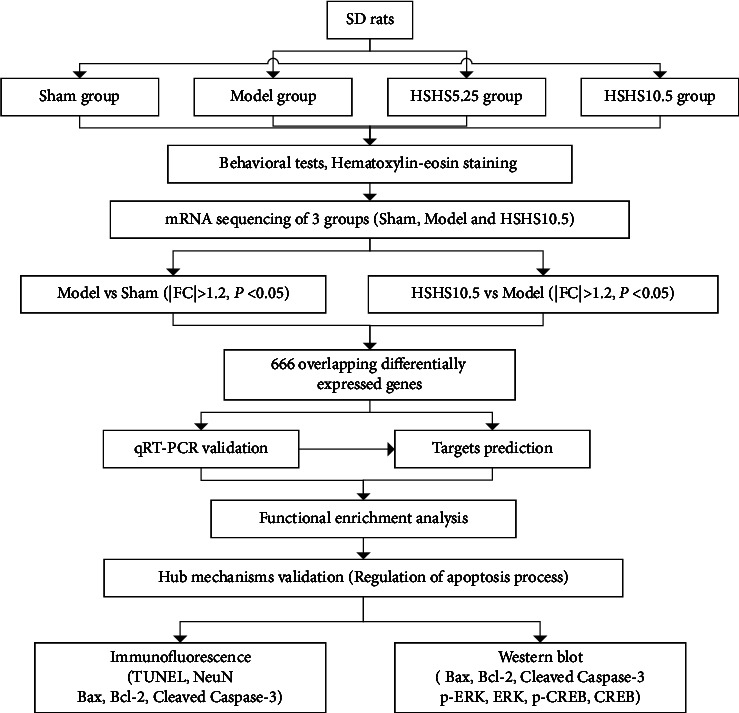
Experimental flow chart.

Herein, we aimed to explore the neuroprotective effect of HSHS on cerebral ischemia in a rat stroke model induced by pMCAO. Furthermore, a deliberate strategy was conducted that integrates transcriptomics methods and experimental verification to investigate the potential mechanisms of HSHS on ischemic stroke.  Figure 1 shows the experimental flow chart of this study.

## 2. Materials and Methods

### 2.1. Animals

In total, forty-eight male Sprague–Dawley rats (280–320 g) were supplied from Beijing Vital River Laboratory Animal Technology Co. Ltd., China. They were kept (three rats/cage) in the specific pathogen-free animal room of the Animal Center of Capital Medical University, China. All animal protocols were approved by the Institution Animal Care and Use Committee of Capital Medical University (No. AEEI-2019-001).

### 2.2. Preparation of Houshiheisan (HSHS)

HSHS formula consists of 13 herbs (Table S1), all obtained from Beijing Tongren-Tang Chinese Medicine Co. Ltd. and authenticated by associate professor Jia Li at Capital Medical University, Beijing. All herbs were mixed and immersed in the 10 × volume of 30% ethanol for 2 h, extracted at 40°C with ultrasound-assisted extraction for 1 h. Afterward, the precipitate was soaked in 8 × 30% ethanol at 40°C with ultrasound-assisted extraction for 40 min. Using a rotary evaporator, the two obtained filtrates were mixed and concentrated into the final extract (1.2 g/mL). Additionally, the chemical compositions of the extract were subjected to quality control [24].

### 2.3. Experiment Design

All rats were randomly separated into four groups after adaptive feeding: the sham, model, HSHS5.25 (HSHS 5.25 g/kg), and HSHS10.5 (HSHS 10.5 g/kg) groups (HSHS 10.5 g/kg was the clinical equivalent daily dose in rats). The pMCAO model was prepared [25]. Rats were anesthetized with isoflurane (5% for induction and 2% for maintenance) in a 2 : 1 N_2_O : O_2_ atmosphere during surgery. After operation for seven days, the ischemic regions of the cortex were frozen in liquid nitrogen and stored at −80°C until further use.

### 2.4. Neurological Functional Assessment

The neurological dysfunction was assessed on postoperative days 1, 3, 5, and 7. The test of neurological deficit was scored as follows [25]: (0) no evident symptoms, (1) unable to fully extend the left forepaw, (2) crawling while spinning to the left side, (3) fall to the left side while crawling, and (4) unable to walk or unconscious.

The beam walking test was used to assess the motor coordination function of rats on days 3 and 7 after pMCAO. Before the operation, each rat was trained to ensure it could habituate to walking on the beam (80 cm long by 3 cm wide, located 60 cm high). The test was scored as follows [26]: (0) cannot stay on the beam, (1) just stay on the beam but not move, (2) try to traverse the beam but fell, (3) traverse the beam with ≥50% hind-limb foot slips, (4) traverse the beam with <50% hind-limb foot slips, (5) traverse the beam and only one hind-limb slip, and (6) traverse the beam with no slips.

### 2.5. Histological Assessment

Seven days after surgery, rats were anesthetized and transcardially perfused with 4% paraformaldehyde. The brains were then routinely embedded in paraffin, sectioned at 4 *μ*m, and stained with HE. Light microscopy was used to observe pathological changes (Nikon, Japan).

### 2.6. Transcript Profile Analysis

Total RNA from the peri-infarct cortex of each group for three rats (sham, model, and HSHS10.5 groups) was extracted with TRIZol reagent (Life Technologies, USA). Then, an RNeasy mini kit (Qiagen, USA) was used to purify total RNA from infarcted tissue. According to Affymetrix protocol, 250 ng total RNA was used to conduct biotinylated cDNA by Ambion® WT Expression Kit. The cDNA fragments were hybridized using a Clariom D assay (rat, Affymetrix) for 16 h at 45°C. Affymetrix Fluidics Station 450 was used to wash and stain GeneChips. All arrays were scanned using GeneChip® Scanner 3000 7G with Affymetrix® GeneChip Command Console (AGCC).

### 2.7. Differentially Expressed Genes (DEGs) Analysis

The moderated F-statistic was used to choose the multigroup DEGs between model vs. sham groups and HSHS10.5 vs. model groups using the R package “limma” (version 3.36.5). *P* values were corrected using limma R Empirical Bayes moderating with Benjamini–Hochberg for multiple test corrections. The threshold set for DEGs was as follows: fold change > 1.2, *P* < 0.05, and false discovery rate (FDR) <0.05. Among sham, model, and HSHS groups, Venn diagrams were used to determine the overlapped DEGs, performed by hierarchical clustering using the R package “heat-map” (version 1.0.12).

### 2.8. Functional Enrichment Analysis

An enrichment analysis of KOBAS-i (https://kobas.cbi.pku.edu.cn/) was performed on the overlapping DEGs using gene ontology (GO) and Kyoto Encyclopedia of Genes and Genomes (KEGG) [27]. The bubble charts of GO and KEGG pathway enrichment were plotted using a free online data analysis and visualization platform (https://www.bioinformatics.com.cn/). Pathways and GO terms were considered markedly enriched at *P* < 0.01.

### 2.9. Quantitative Real-Time PCR Validation

The total RNA of the ischemic cortex was obtained with a TRIZol reagent (Life Technologies, USA). Real-time PCR was conducted using a one-step qRT-PCR kit (Toyobo, Japan) and quantified using the Bio-Rad CFX with a 20 *μ*L system (Bio-Rad, United States). Relative quantification of mRNAs was performed using the ^2−ΔΔ^Ct method, and each sample was normalized.  Table S2 lists the PCR primers.

### 2.10. TUNEL Assay

The paraffin slices were dewaxed and hydrated. Brain slices were washed in PBS containing proteinase K (20 *μ*g/mL), and stained with TUNEL detection reagent (G1501, Servicebio, China) at 37°C for 1 h. The sections were collected using a fluorescent microscope (Nikon, Japan). ImageJ was utilized to quantify the number of TUNEL-positive cells.

### 2.11. Immunofluorescence Analysis

The brain sections were incubated with rabbit anti-NeuN (1 : 400, Cat no. 66836-1-Ig, ProteinTech, United States (US)), rabbit anti-Bax (1 : 400, Cat no.50599-2-Ig, ProteinTech, US), and rabbit anti-Bcl-2 (1 : 400, Cat no.12789-1-AP, ProteinTech, US) or rabbit anticleaved caspase-3 (1 : 400, #9664, CST, US), respectively, at 4°C overnight. Afterward, the sections were supplied with FITC (1 : 400, ZSGB-BIO, China) or Cy3 (1 : 400, Beyotime, China), incubated for 2 h, and stained with DAPI (SouthernBiotech, US). Sections were collected using a fluorescent microscope (Nikon, Japan). Five fields of view were randomly selected from each section, and the average value of integrated optical density was calculated using ImageJ software.

### 2.12. Western Blotting

The protein sample with  × 5 loading buffer was boiled, electrophoresed on a 12% polyacrylamide gel, and transferred to PVDF membranes. Membranes were blocked with 5% skim milk or bovine serum albumin dissolved in Tris-buffered saline with 0.1% Tween-20 (TBST) for 1 h, and incubated overnight at 4°C with rabbit anti-Bax (Cat No. 12789-1-AP, 1 : 5000, ProteinTech, US), rabbit anti-Bcl-2 (Cat no. 12789-1-AP, 1 : 2000, ProteinTech, US) or rabbit anti-cleaved caspase-3 (#9664, 1 : 1000, CST, US), anti-p-ERK1/2 (#4370, 1 : 1000, CST, US), anti-ERK1/2 (#4695, 1 : 1000, CST, US), anti-p-CREB (1 : 1000, #9198, CST, US), anti-CREB (#9197, 1 : 1000, CST, US) or anti-tubulin (GTX101279, 1 : 40000, GeneTex, US), and mouse anti-GAPDH (GTX627408, 1 : 10000, GeneTex, US) at 4°C, respectively. The next day, washed three times for 10 min in TBST, the membranes were incubated with appropriate secondary antibody for 1 h, and washed for another three times for 10 min at room temperature. Immunoreactive bands were observed with the enhanced chemiluminescence detection reagent (Millipore, USA) and analyzed using ImageJ software.

### 2.13. Data Analysis and Statistics

Results were expressed as mean ± standard error (SEM) and analyzed using GraphPad Prism 8.0.2 software. The comparison of data between groups was analyzed using one-way analysis of variance (ANOVA) with the least significant difference test for multiple comparisons. Statistical significance was defined as *P* < 0.05.

## 3. Results

### 3.1. HSHS Improved Neurological Deficits and Pathological Injury in pMCAO Rats

**Figure 2:**
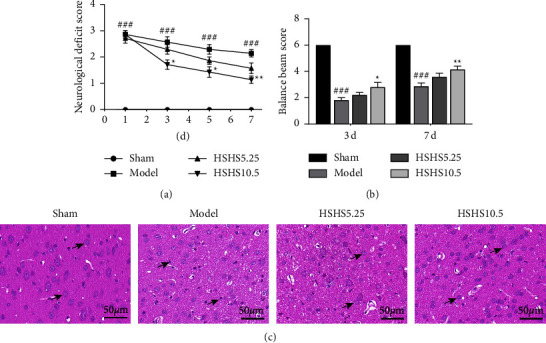
Effect of HSHS on neurological deficits and pathological damage in pMCAO rats: (a) neurological deficit scores from d 1, 3, and 5∼7 after the operation; (b) balance beam scores at d 3 and d 7 after the operation; (c) HE staining in the peri-infarct cortex 7 d after pMCAO (arrows: neuron). Results were presented as mean ± SEM. *n* = 7, ^###^*P* <0.001 vs. sham group, and ^*∗*^*P* < 0.05 and ^*∗∗*^*P* < 0.01 vs. model group.

To assess the neuroprotective effect of HSHS on pMCAO rats, neurological tests and hematoxylin and eosin (HE) staining were conducted. Compared to the model group, neurological deficit scores of HSHS10.5 group rats were reduced on days 3 and 5∼7 after the operation (*P* < 0.05 or *P* < 0.01), while the HSHS5.25 group showed a nonsignificant decrease (Figure 2(a)). The beam walking test suggested that rats in the treated group performed a better motor function after pMCAO. The balancing beam scores in the model group decreased compared to the sham group on the 3^rd^ and 7^th^ day (*P* < 0.001) (Figure 2(b)). Compared to the model group, the scores in HSHS10.5 group increased on the 3^rd^ and 7^th^ day (*P* < 0.05 or *P* < 0.01). Balance beam scores in the HSHS5.25 group increased, but with a nonsignificant difference.

HE staining revealed that neurons were disorderly arranged, the cell membrane was vague, the cell body was shrunk, the nucleus was stained with pyknosis, and neurons were missing in the ischemic brain. The treatment with HSHS decreased the pathological abnormalities of the ischemic brain in pMCAO rats (Figure 2(c)).

### 3.2. HSHS Altered Gene Expression Profiles in pMCAO Rats

**Figure 3:**
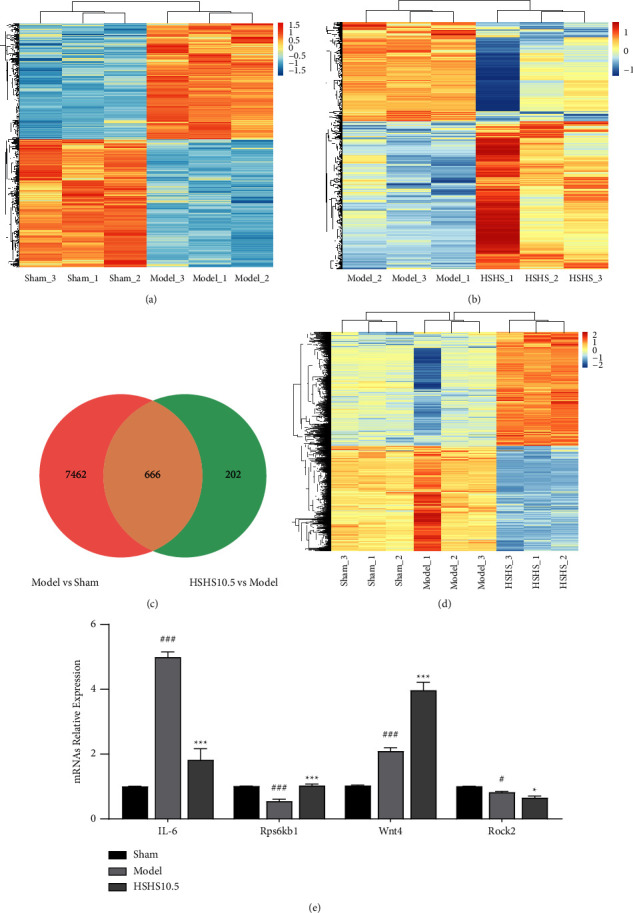
HSHS altered gene expression profiles in pMCAO rats. The hierarchical clustering of the sham and model groups (a) and the model and HSHS (HSHS10.5) groups (b). Downregulated genes are shown in blue, and upregulated genes are shown in red (*P* < 0.05). (c) The Venn diagram showed the total number of upregulated and downregulated genes in the peri-infarct cortex of the sham, model, and HSHS groups. (d) The heat-map among the three groups showed the significantly changed genes. (e) Validation of the microarray results by qPCR. Results were presented as mean ± SEM, *n* = 4. ^#^*P* < 0.05 and ^###^*P* < 0.001 vs. sham group, and ^*∗*^*P* < 0.05 and ^*∗∗∗*^*P* < 0.001 vs. model group.

To further investigate the molecular mechanisms of HSHS, gene expression profiles in pMCAO rats were analyzed using high-throughput microarray technology. In total, 8128 DEGs were identified in the cortex of pMCAO rats between the model and sham group (Figure 3(a)). There were 868 DEGs in the cortex of pMCAO rats between the HSHS10.5 and model groups (Figure 3(b)). We obtained 666 DEGs that overlap for further analysis using the Venn plot (Figures 3(c) and 3(d)). The results of qRT-PCR confirmed the reliability of microarray data (Figure 3(e)).

### 3.3. Functional Enrichment Analysis of DEGs

**Figure 4:**
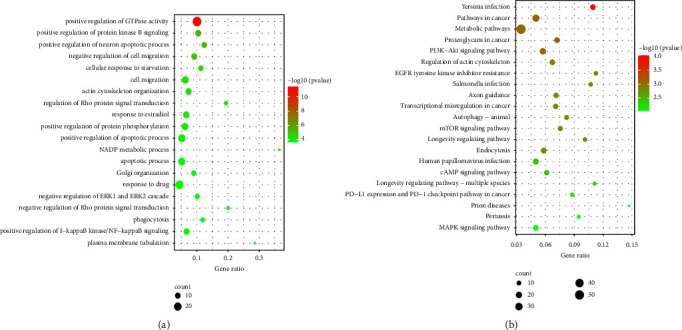
Functional enrichment analysis of DEGs: (a) the top 20 significant biological processes and (b) the top 21 significant KEGG pathways.

We imported the 666 DEGs into the KOBAS-i database for GO and KEGG pathway analyses to explore functional distribution in the DEGs. These genes were associated with multiple biological processes (BP) (*P* < 0.01) (Figure 4(a)). BP terms were mainly enriched in positive regulation of the neuronal apoptotic process, positive regulation of the apoptotic process, apoptotic process, and negative regulation of the ERK1 and ERK2 cascade, suggesting that HSHS exerts beneficial effects on ischemic stroke by regulating the apoptotic process.

Pathway annotation suggested that these genes were involved in 50 pathways (*P* < 0.01). The top 21 pathways were performed (Figure 4(b)). Among these pathways, the PI3K/AKT signaling, mTOR signaling, and MAPK signaling pathways were highly associated with the target genes.

### 3.4. HSHS Prevented Neuronal Apoptosis in pMCAO Rats

**Figure 5:**
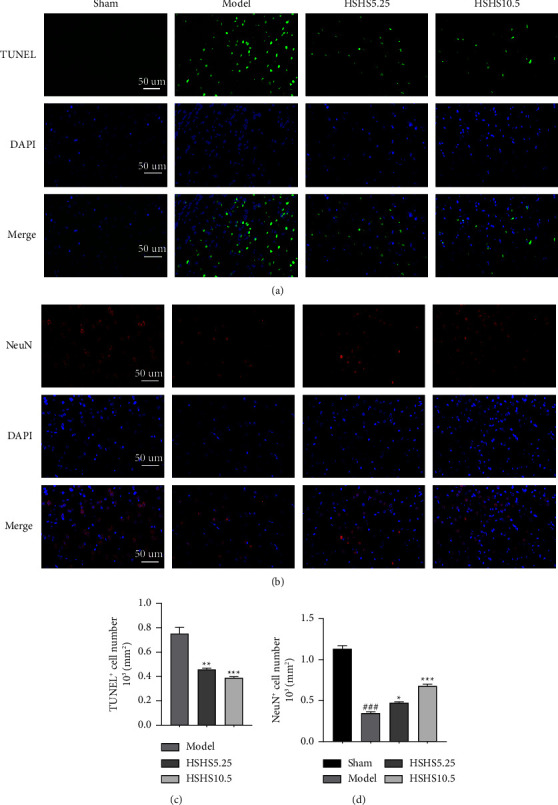
Effect of HSHS on neuronal apoptosis in pMCAO rats. (a, b) Micrographs of the TUNEL-labeled and NeuN-labeled in the peri-infarct cortex. (c) Quantitative data of apoptotic cells in various groups. (d) Quantitative data of the NeuN^+^ cells in various groups of rats. Scale bars: 50 *μ*m and magnification: 400×. Results were presented as mean ± SEM, *n* = 3. ^###^*P* < 0.001 vs. sham group, and ^*∗*^*P* < 0.05, ^*∗∗*^*P* < 0.01 and ^*∗∗∗*^*P* < 0.001 vs. model group.

In pMCAO rats, the peri-infarct cortex cell apoptosis was evaluated by TUNEL staining. The model group showed significantly more apoptotic cells that emit green fluorescence than the sham group, while the number of apoptotic neuronal cells in the HSHS5.25 and HSHS10.5 groups was significantly reduced compared to the model group (*P* < 0.01, *P* < 0.001) (Figures 5(a) and 5(c)). The immunofluorescence method measured neuronal-specific marker NeuN (Figure 5(c)). According to the quantitative analysis, NeuN immunoreactivity in the model group significantly decreased (*P* < 0.001) (Figure 5(d)). The number of NeuN-positive cells significantly increased in HSHS5.25 (*P* < 0.05) and HSHS10.5 groups (*P* < 0.001) compared to the model group (Figure 5(d)).

### 3.5. HSHS Regulated Apoptosis-Related Proteins in pMCAO Rats

**Figure 6:**
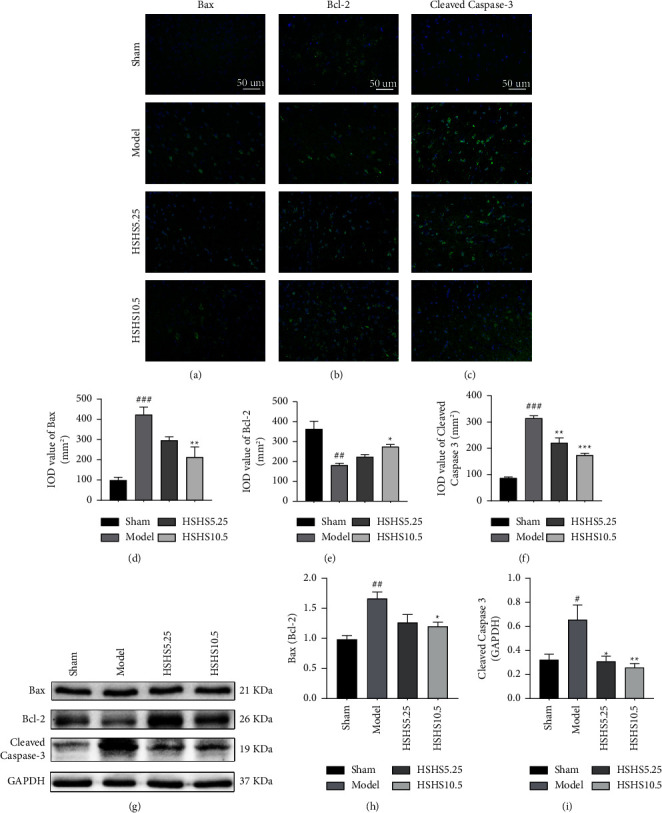
Effect of HSHS on regulated apoptosis-related proteins in pMCAO rats. The representative images and immunofluorescence analysis for Bax (a, d), Bcl-2 (b, e), and cleaved caspase-3 (c, f) in the peri-infarct cortex (*n* = 3). (g–i) The protein levels of Bax, Bcl-2, and cleaved caspase-3 were determined by western blot analysis (*n* = 4). Results were presented as mean ± SEM. ^#^*P* < 0.05, ^##^*P* < 0.01, and ^###^*P* < 0.001 vs. sham group, and ^*∗*^*P* < 0.05, ^*∗∗*^*P* < 0.01, and ^*∗∗∗*^*P* < 0.001 vs. model group.

Bax, Bcl-2, and cleaved caspase-3 are major apoptosis-related proteins. The expressions of Bax, Bcl-2, and cleaved caspase-3 were examined using immunofluorescence (Figures 6(a)–6(c)). The expressions of Bax and cleaved caspase-3 in the peri-infarct cortex were markedly upregulated compared to that of the model group (*P* < 0.001) (Figures 6(d) and 6(e)), but the expression of Bcl-2 was significantly downregulated (*P* < 0.01) (Figure 6(f)). In pMCAO rats that received HSHS10.5 treatment, the expressions of Bax (*P* < 0.01) and cleaved caspase-3 (*P* < 0.001) were significantly elevated, and Bcl-2 expression was increased (*P* < 0.05) in the peri-infarct cortex compared to the model group. Cleaved caspase-3 expression in the peri-infarct cortex was reduced in the HSHS5.25 group compared to the model group (*P* < 0.01). Furthermore, the cortex around the infarction was examined using a western blot to confirm the regulation of HSHS for apoptosis-related proteins in pMCAO rats (Figures 6(g)–6(i)). We observed a significant increase in the Bax/Bcl-2 ratio in the model group compared to the sham group (*P* < 0.01) and a substantial decrease after HSHS10.5 treatment (*P* < 0.05) (Figure 6(h)). Furthermore, rats treated with HSHS5.25 and HSHS10.5 showed less cleaved caspase-3 protein level than that of the model group (*P* < 0.05, *P* < 0.01) (Figure 6(i)), consistent with the result of immunofluorescence.

### 3.6. HSHS Increased Expression of ERK1/2-CREB Signaling Pathway-Related Proteins in pMCAO Rats

**Figure 7:**
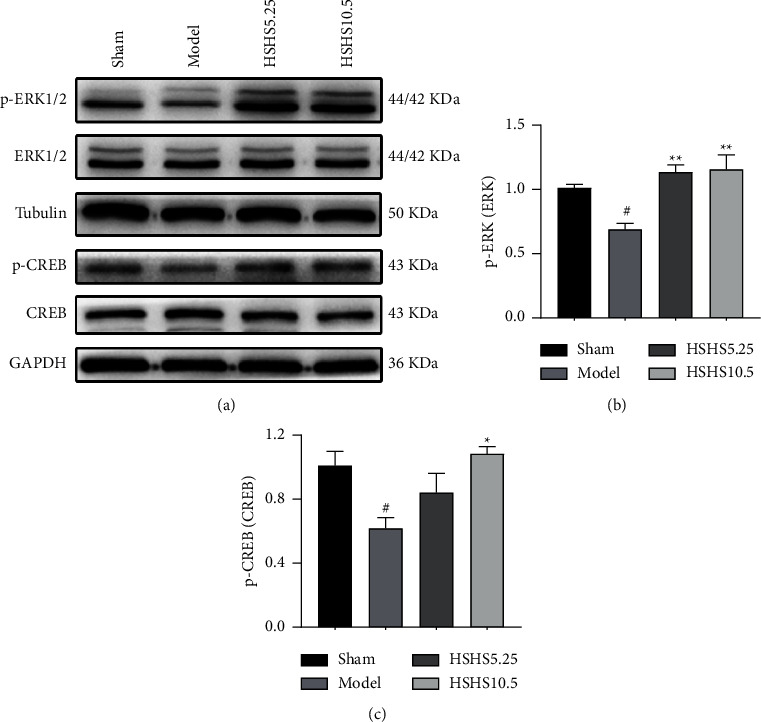
Effect of HSHS on the expression of ERK1/2-CREB pathway-related proteins in pMCAO rats. (a) The representative images of p-ERK1/2 (p-ERK), ERK1/2 (ERK), p-CREB, and CREB. The western blot analysis of p-ERK/ERK (b) and p-CREB/CREB (c). Results were presented as mean ± SEM, *n* = 3. ^#^*P* < 0.05 vs. sham group, and ^*∗*^*P* < 0.05 and ^*∗∗*^*P* < 0.01 vs. model group.

tThe expressions of ERK1/2-CREB signaling-related proteins were examined to further investigate the possible mechanisms of HSHS on ischemic stroke using western blot. Compared to the sham group, the p-ERK1/2/ERK1/2 ratio in the model group was downregulated (*P* < 0.05). The rats in the HSHS5.25 and HSHS10.5 groups showed a significant increase in the p-ERK1/2/ERK1/2 ratio compared to the model group (*P* < 0.01) (Figures 7(a) and 7(b)). Compared to the sham group, the p-CREB/CREB ratio in the model group was downregulated (*P* < 0.05), while the p-CREB/CREB ratio in the HSHS10.5 group increased compared to the model group (*P* < 0.05) (Figures 7(a) and 7(c)). The p-CREB/CREB ratio in the HSHS5.25 group was also upregulated (Figure 7(c)).

## 4. Discussion

Ischemic stroke is mainly a disorder of blood supply to the brain resulting from various causes, contributing to a clinical syndrome characterized by hypoxic-ischemic damage to brain tissue [28]. Besides vascular recanalization, TCM has shown remarkable neuroprotective effects and gained great attention in treating ischemic stroke [29]. In TCM, HSHS was created by Zhang et al. to treat stroke, following the pathogenesis of deficiency of genuine qi and excess of pathogenic factor [12]. Furthermore, HSHS has been used to treat stroke for approximately 2000 years and is safe and effective. However, the molecular mechanism of action has not yet been fully elucidated. Herein, transcriptome analysis and *in vivo* experiments were used to systematically investigate the pharmacological mechanisms of HSHS in treating ischemic stroke.

Herein, HSHS exerted neuroprotective activity on pMCAO rats by improving the symptoms of neurological impairment and pathological injury. Then, the high-throughput sequencing technology of the microarray chip was conducted to explore the therapeutic mechanism of HSHS for ischemic stroke from the whole transcriptome level. We identified 8128 DEGs between the model and sham groups and 868 DEGs between the HSHS10.5 and model groups. We obtained 666 intersection DEGs between the sham, model, and HSHS10.5 groups. Furthermore, GO enrichment analysis on total intersection DEGs showed that the effects of HSHS on ischemic stroke were associated with positive regulation of neuron apoptotic process, positive regulation of the apoptotic process, apoptotic process, and negative regulation of ERK1 and ERK2 cascade. Moreover, the KEGG pathways analysis demonstrated that the intersection of DEGs was mainly associated only with the PI3K-Akt signaling, mTOR signaling, and MAPK signaling pathways. Accordingly, the neuroprotective effect of HSHS in stroke rats was related to the regulation of neuronal apoptosis.

Apoptosis plays a vital role in ischemia-induced neuronal death in ischemic stroke [30]. In the infarct core, excitotoxicity and neuronal necrosis occur in several minutes [31]. However, many dormant or semidormant nerve cells in the ischemic penumbra mainly occur in delayed death in the form of apoptosis [32]. These cells are the most possible and valuable to be rescued in clinics [30]. Subsequently, preventing neuronal apoptosis in the penumbra and improving its dysfunction is vital to treat ischemic stroke [33]. Herein, TUNEL and NeuN staining results showed that HSHS reduced the number of cell apoptosis to protect neurons in pMCAO rats. Meanwhile, treatment of HSHS decreased the expression of proapoptotic proteins Bax and cleaved caspase-3 and increased antiapoptotic protein Bcl-2 expression. The previous results indicated that HSHS significantly increased the number of surviving neurons by preventing apoptosis in the peri-infarct of pMCAO rats.

We evaluated the related signaling pathways and found that HSHS could suppress apoptosis by activating the ERK1/2-CREB signaling pathway. Mitogen-activated protein kinase (MAPK) signaling pathway controls different physiological processes, such as cell growth, development, division, and death [34, 35]. As a member of the MAPK family, extracellular regulated protein kinases (ERKs) exert a crucial role in transmitting signals from surface receptors to the nucleus. Phosphorylated ERKs are transferred from the cytoplasm to the nucleus to regulate cell proliferation, survival, differentiation, and apoptosis [34, 36]. Upregulating ERK1/2 pathway activity is associated with neuronal survival in ischemic stroke models *in vivo* and *in vitro* [37, 38]. ERK1/2 acts as a neuroprotective agent by inhibiting postischemic oxidative stress and mitochondria-dependent apoptosis of neural cells [39, 40]. ERK1/2 activation can promote the phosphorylation of CREB, increase the expression of prosurvival protein Bcl-2, inhibit ischemia-induced neuronal apoptosis, and enhance neuronal survival [41, 42]. As a post-translationally activated transcription factor, cyclic AMP response element binding protein (CREB) participates in many brain functions, such as promoting neuronal survival mainly by increasing the expression of neurotrophic factors and antiapoptotic genes [43, 44]. Hypoxia and ischemia increase the phosphorylation of CREB in brain tissue. However, inhibiting the phosphorylation of CREB reduces Bcl-2 expression [45]. Herein, the phosphorylation of ERK1/2 and CREB was downregulated in pMCAO rats, whereas HSHS treatment protected neurons and increased ERK1/2 and CREB phosphorylation. These results indicated that ERK1/2-CREB pathway activation might play a vital role in the neuroprotection of HSHS on ischemic stroke. These findings provide a solid theoretical basis for the clinical application of HSHS in ischemic stroke.

## 5. Conclusion

This study used transcriptome analysis and in vivo experiments to systematically investigate the neuroprotective mechanisms of HSHS in ischemic stroke. The results suggested that HSHS may prevent neuronal apoptosis by activating the ERK1/2-CREB signaling pathway, providing a novel insight for treating ischemic stroke.

## Data Availability

The data that support the findings of the study are available in this article.

## Supplementary Materials

Supplementary Materials Table S1: herbal formula of Houshiheisan. Table S2: sequence of primers for qPCR.
