# Supplementary material for: Study on Neuroprotective Mechanism of Houshiheisan in Ischemic Stroke Based on Transcriptomics and Experimental Verification
Source: Evid Based Complement Alternat Med. 2023 Feb 6;2023:8673136. doi: 10.1155/2023/8673136 (PMC9925249; doi:10.1155/2023/8673136)
Supplement: Supplementary Materials — Table S1: herbal formula of Houshiheisan. Table S2: sequence of primers for qPCR. [file 8673136.f1.zip › Table S1.docx]

T_ABLE_ S1: Herbal formula of HSHS.

| Latin Name of the Medicinal Material | Chinese Name | English name | Family | Part used | Weight  [g] |
| --- | --- | --- | --- | --- | --- |
| *Flos Chrysanthemi* | Ju Hua | Chrysanthemum morifolium Ramat. | Compositae | flos | 40 |
| *Rhizoma Atractylodis Macrocephalae* | Bai Zhu | Atractylodes macrocephala Koidz. | Compositae | rhizomes | 10 |
| *Radix et Rhizoma Asari* | Xi Xin | Asarum heterotropoides Fr. Schmidt var. mandshuricum (Maxim.) Kitag. | Aristolochiaceae | roots and rhizomes | 3 |
| *Poria* | Fu Ling | Poria cocos（Schw.）Wol£ | Polyporaceae | sclerotium | 3 |
| *Ostreae Concha* | Mu Li | Ostrea gigas Thunberg | Ostreidae | shell | 3 |
| *Radix Platycodonis* | Jie Geng | Platycodon grandiflorum （Jacq.）A.DC. | Campanulaceae | root | 8 |
| *Radix Saposhnikoviae* | Fang Feng | Saposhnikovia divaricate （Turcz.）Schischk. | Umbelliferae | root | 10 |
| *Radix et Rhizoma Ginseng* | Ren Shen | Panax ginseng C. A. Mey. | Araliaceae | roots and rhizomes | 3 |
| *Rhizoma Chuanxiong* | Chuan Xiong | Ligusticum chuanxiong Hort | Umbelliferae | rhizomes | 3 |
| *Radix Scutellariae* | Huang Qin | Scutellaria baicalensis Georgi | Lamiaceae | root | 5 |
| *Radix Angelicae Sinensis* | Dang Gui | Angelica sinensis（Oliv.）Diels | Umbelliferae | root | 3 |
| *Rhizoma Zingiberis* | Gan Jiang | Zingiber officinale Rosc. | Zingiberaceae | rhizomes | 3 |
| *Ramulus Cinnamomi* | Gui Zhi | Cinnamomum cassia Presl | Lauraceae | ramulus | 3 |
