# Supplementary material for: Study on Neuroprotective Mechanism of Houshiheisan in Ischemic Stroke Based on Transcriptomics and Experimental Verification
Source: Evid Based Complement Alternat Med. 2023 Feb 6;2023:8673136. doi: 10.1155/2023/8673136 (PMC9925249; doi:10.1155/2023/8673136)
Supplement: Supplementary Materials — Table S1: herbal formula of Houshiheisan. Table S2: sequence of primers for qPCR. [file 8673136.f1.zip › Table S2.docx]

T_ABLE_ S2: Sequence of primers for qRT-PCR.

| Gene | forward | reverse |
| --- | --- | --- |
| IL-6 | 5’-AGGATACCACCCACAACAGACC-3’ | 5’-TTGCCATTGCACAACTCTTTTC-3’ |
| Wnt4 | 5’-ATCCTGACACACATGCGGGT-3’ | 5’-ATCCGTATGTGGCTTGAACTGC-3’ |
| Rock2 | 5’-TGCTATTGGATAAACACGGACA-3’ | 5’-ACCAATCACATTCTCGTCCATAG-3’ |
| Rps6kb1 | 5’-GAAATGCTGCTTCTCGTCTTGG-3’ | 5’-AGACCTGGTTGGCACTTTCACT-3’ |
| GAPDH | 5’-CTGGAGAAACCTGCCAAGTATG-3’ | 5’-GGTGGAAGAATGGGAGTTGCT-3’ |
